# Modulation of Vestibular Microphonics: A Historical Note

**DOI:** 10.3390/audiolres11030036

**Published:** 2021-08-06

**Authors:** Hero P. Wit

**Affiliations:** 1 Department of Otorhinolaryngology/Head and Neck Surgery, University Medical Center Groningen, University of Groningen, 9712 GZ Groningen, The Netherlands; h.p.wit@umcg.nl; 2 Graduate School of Medical Sciences (Research School of Behavioural and Cognitive Neurosciences), University of Groningen, 9712 GZ Groningen, The Netherlands

**Keywords:** vestibular organ, hair cells, mechano-electric transduction, rotational stimuli

## Abstract

Modulation of microphonics has recently been used to investigate the sensitivity of the utricle in the vestibular organ of the guinea pig. The same technique was used more than 30 years ago to obtain information on the processing of rotational stimuli in the horizontal semicircular canals of the pigeon. Data from that time were reanalysed to give a relation that describes the mechano-electrical transduction (MET) process in vestibular hair cells.

## 1. Introduction

One of the experimental techniques that has recently been used by Pastras et al. [1] to investigate the sensitivity of the guinea pig utricle to bone-conducted vibration is monitoring utricular microphonics during low-frequency hydrodynamic biasing of the position of the utricular macula. This reminded the author of the present note of work that was done more than 30 years ago [2], and where the experimental method was rotational modulation of the semicircular canal microphonics, evoked by air-conducted sound stimuli [3,4]. Vestibular microphonics (VM) have been recorded using a wide array of stimuli, in several mammalian and non-mammalian models [5]. It provides an objective measure of vestibular hair cell function in various experimental animals models [2,5]. Like the cochlear microphonic, the technique also has the ability to differentially diagnose peripheral nerve from hair cell dysfunction.

The first publication that proved the existence of vestibular microphonics (cochlear microphonics were discovered 20 years earlier [6]) appeared in 1949 [3]. It was the result of a collaboration between De Vries from the Physics Laboratory of the University of Groningen (The Netherlands) and Bleeker from the ENT Department of the University Clinic of Groningen. This started a long period of biophysical research of the vestibular system in this clinic (now called: Department of Otorhinolaryngology/Head and Neck Surgery of the University Medical Center Groningen), during which—among many other things—modulation of microphonics by (natural) rotational stimulation was explored as a method to obtain information on the mechano-electrical transduction (MET) in vestibular hair cells.

Natural stimuli for the vestibular system are mainly in the very low frequency range (below 10 Hz). However, the vestibular system is also sensitive to air-conducted sound [7,8,9,10] and to bone-conducted vibration [5], in the 0.5–5 kHz frequency range. Air-conducted sound in this frequency range evokes microphonic responses in the peripheral vestibular system [4]. However, due to the lowpass filter characteristics of vestibular hair cells, the AC component of the vestibular microphonic is largely attenuated at the hair cell corner frequency around 1–2 kHz [11].

This current work reanalyses data from the old study showing the rotational modulation of microphonics in pigeons. Results demonstrate a reliable computational approach to approximate peripheral vestibular sensitivity and MET channel gating from the semi-circular canals, analogous to the recent work by Pastras et al. [1].

## 2. Materials and Methods

**Figure 1:**
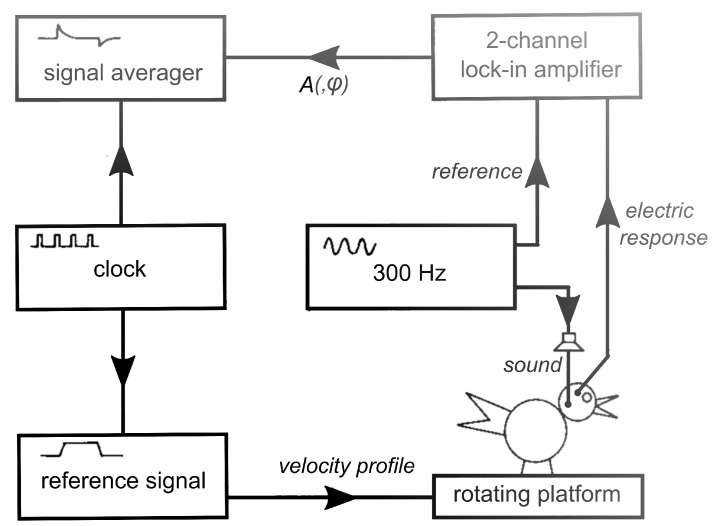
Schematic of stimulation and recording equipment. The clock synchronises signal averager and reference signal generator. The lock-in amplifier is used in the amplitude-phase (A,ϕ) mode. (Modified from Reference [2]).

For the original paper [2], presented at the *Bárány Society Meeting* in Bologna (1987), experiments were performed in more than 20 homing pigeons (Columba livia), from which the membraneous cochleas were removed more than 4 weeks prior to an experiment. This operation slightly affects the vestibular system, as was learned from recordings of microphonics performed too soon after cochlea extirpation, but within 4 weeks, complete recovery takes place. The following methodology was taken from Wit et al. [2]: The bird was placed on an electronically controlled rotating platform with its lateral canals in a horizontal plane and anesthetized with halothane (1%) in oxygen (1 L/min). A 300 Hz constant amplitude pure tone (70 dB SPL) was delivered to the vestibular system via ear canal and columella, through a silastic tube. (See Wit et al. [10] for the choice of this frequency). A small hole was cut in the bony wall of the lateral canal. This fenestration does not affect the membraneous part of the vestibular system, while creating an artificial round window makes the vestibular system more sensitive to sound. The vestibular microphonics potential, evoked by the continuous pure tone stimulus, was measured with differential thin wire electrodes. One electrode contacted perilymph through a small hole in the top of the right horizontal ampulla; the other was placed in a hole in the vestibulum. The amplitude of the microphonics potential was measured with a two-phase lock-in analyzer (EG&G 5206) and recorded with a signal averager (Datalab DL 4000), as shown in Figure 1. The platform was rotated with a sinusoidal or with a trapezoidal velocity profile. The profile was symmetric around velocity zero. As a rule, responses to eight full rotational cycles were averaged.

## 3. Analysis of a Result

**Figure 2:**
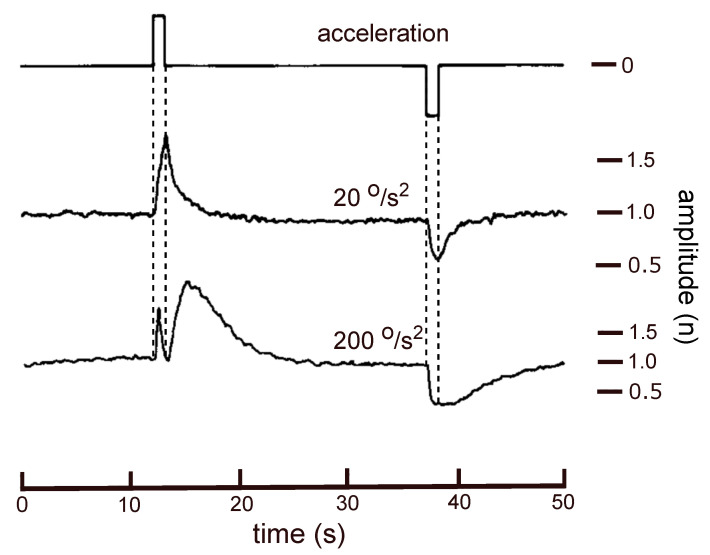
Upper trace: Acceleration (1 s rectangular pulses) during one rotational cycle with a trapezoidal velocity profile. Middle and lower trace: Averaged amplitude of the microphonics potential on a normalised scale, for acceleration pulses of 20 and 200 ${}^{0}/s^{2}$ respectively. (Modified from Reference [2]).

With a trapezoidal velocity profile sudden changes are created in the amplitude of the microphonics signal, followed by an exponential decay. For high acceleration values a sharp extra minimum, coinciding with the offset of the positive acceleration pulse, appears in the response amplitude (Figure 2, lower trace).

No numerical data from the original experiments, performed more than 30 years ago, are left. Therefore, the middle part of the lower trace in Figure 2 was digitised with *PlotDigitizer*. The result is shown with the thick grey line in Figure 4.

Géléoc et al. [12] measured mechano-electrical transduction (MET) in vestibular hair cells (and in cochlear outer hair cells) in organotypic cultures from neonatal mice, by displacing their stereociliary bundles with a fluid jet. They fitted their data with the following relation between transducer current *i* and bundle displacement *x*:

(1) $$i=a/\left(1+K_{2}\left(1+K_{1}\right)\right);K_{1,2}=e^{\alpha_{1,2}\left(x_{1,2}-x\right)}$$

**Figure 3:**
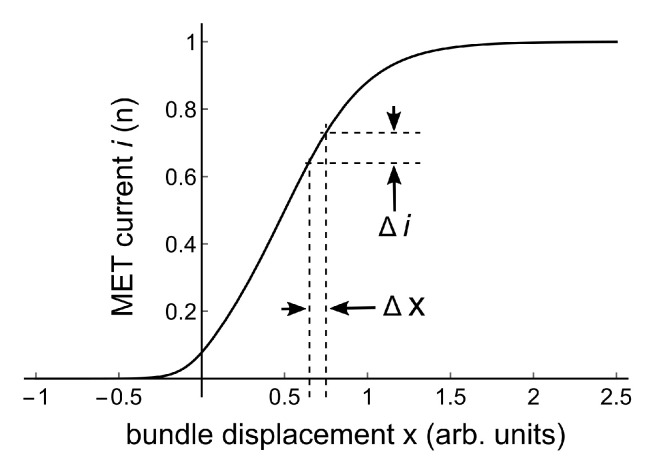
Solid line: MET-curve for parameters *a* = 1, $x_{1}$ = −0.05, $x_{2}$ = 0.5, $\alpha_{1}$ = 10, $\alpha_{2}$ = 4. A small bundle displacement Δx creates a small MET current change Δi .

$K_{1,2}$ are equilibrium constants of transitions between transducer channel states. The set points for these transitions are at displacements $x_{1,2}$, and $\alpha_{1,2}$ are transition sensitivities. Figure 3 gives an example of this MET-curve for a chosen set of parameters.

If the continuous 300 Hz constant amplitude pure tone creates a small constant bundle displacement Δx, the MET current will change with an amount Δi, as illustrated in Figure 3. The measured microphonics amplitude will be proportional to the derivative di/dx of a single MET-curve if the following assumptions are made: 1. All hair cells have the same MET-curve; 2. The contribution of a particular hair cell to the microphonics amplitude is proportional to Δi; 3. Δx is small. This derivative is the slope *s* of the MET-curve. As a function of bundle position *x* it is given by:

(2) $$s\left[x\right]=\frac{ae^{\alpha_{2}\left(x_{2}-x\right)}\left(\alpha_{2}+\left(\alpha_{1}+\alpha_{2}\right)e^{\alpha_{1}\left(x_{1}-x\right)}\right)}{{\left(1+e^{\alpha_{2}\left(x_{2}-x\right)}+e^{\alpha_{1}x_{1}+\alpha_{2}x_{2}-\left(\alpha_{1}+\alpha_{2}\right)x}\right)}^{2}}$$

An acceleration pulse (Figure 2) pushes the hair bundles from their resting position during 1 s. After which they will return to this position during the 24 s constant velocity intervals of the rotational stimulus. It is supposed that this return slows down and is given by $x\left[t\right]=e^{-\beta t}$, (β is a constant, *t* is time).

**Figure 4:**
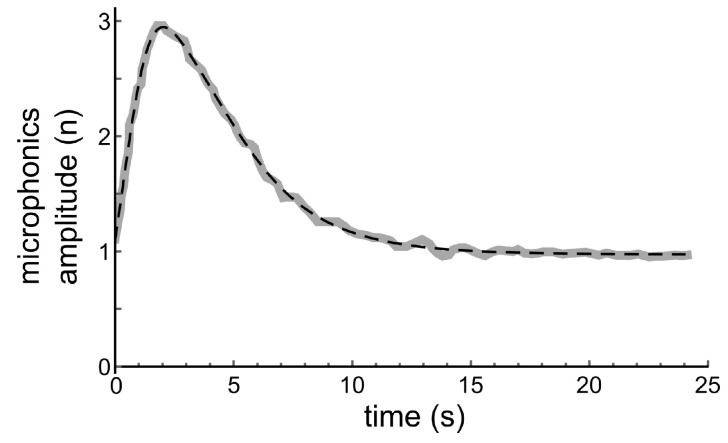
Thick grey line: Digitised middle part of the lower trace in Figure 2. Dashed line: Fit with Equation (2) for the relation between microphonics amplitude and hair bundle displacement, assuming an exponentially decreasing velocity for the return of the hair bundle to its resting position. Fit parameters are $\alpha_{1}$ = 8.77, $\alpha_{2}$ = 4.32, $x_{1}$ = 0.0552, $x_{2}$ = 0.514, β = 0.363.

After replacement of *x* in Equation (2) with x[t], a perfect fit with s[x] to the solid curve in Figure 4 could be found with *Mathematica*’s FindFit-routine, for parameters *a* = 2.62, $\alpha_{1}$ = 8.77, $\alpha_{2}$ = 4.32, $x_{1}$ = 0.0552, $x_{2}$ = 0.514, β = 0.363; as is shown with the dashed line in this figure.

**Figure 5:**
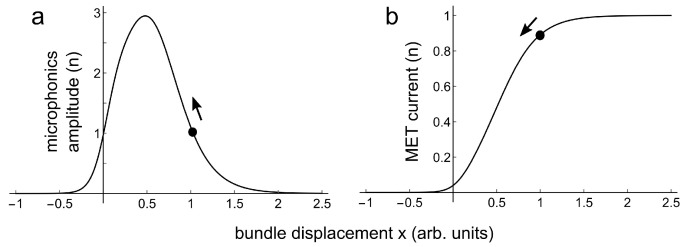
(**a**) Relation between bundle displacement and microphonics amplitude for parameters *a* = 2.62, $\alpha_{1}$ = 8.77, $\alpha_{2}$ = 4.32, $x_{1}$ = 0.0552, $x_{2}$ = 0.514, β = 0.363. (**b**) Relation between bundle displacement and MET current for the same values of $\alpha_{1},\alpha_{2},x_{1},x_{2}$. The black dots in (**a**,**b**) are at corresponding bundle displacement starting positions. The arrows indicate the direction along curves (**a**,**b**) of the return of microphonics amplitude and MET current to their equilibrium values.

Figure 5a is a plot of Equation (2), for the same values for $a,\alpha_{1},\alpha_{2},x_{1},x_{2}$ as for the fit in Figure 4. The black dots in this figure indicate bundle displacement right after the positive going acceleration pulse, being at time zero in Figure 4. From there the bundle travels back to its equilibrium position, as indicated with the arrows. Figure 5b is a plot for Equation (1), again for these values for $\alpha_{1},\alpha_{2},x_{1},x_{2}$, and for a=1.

## 4. Conclusions

This paper describes an—at that time—novel technique to approximate MET channel conductance and VM sensitivity during longitudinal experimental manipulations, similar to recent work by Pastras et al. [1]. The results in the original conference contribution [2] were explained by assuming a sigmoidal relation between hair cell conductance and hair bundle deviation. In the present note one of the results from that time is reanalysed along the same line as this was done originally, but now with an experimentally obtained relation between hair cell conductance and hair bundle deviation [12], that was not yet available at the time of the original work.

## Data Availability

Not applicable.
